# Myeloid deletion of talin-1 reduces mucosal macrophages and protects mice from colonic inflammation

**DOI:** 10.1038/s41598-023-49614-z

**Published:** 2023-12-15

**Authors:** Yvonne L. Latour, Kara M. McNamara, Margaret M. Allaman, Daniel P. Barry, Thaddeus M. Smith, Mohammad Asim, Kamery J. Williams, Caroline V. Hawkins, Justin Jacobse, Jeremy A. Goettel, Alberto G. Delgado, M. Blanca Piazuelo, M. Kay Washington, Alain P. Gobert, Keith T. Wilson

**Affiliations:** 1 Department of Pathology, Microbiology, and Immunology, Vanderbilt University Medical Center, 2215B Garland Ave., 1030C MRB IV, Nashville, TN 37232-0252 USA; 2 Division of Gastroenterology, Hepatology, and Nutrition, Department of Medicine, Vanderbilt University Medical Center, Nashville, TN USA; 3 Program in Cancer Biology, Vanderbilt University School of Medicine, Nashville, TN USA; 4 Center for Mucosal Inflammation and Cancer, Vanderbilt University Medical Center, Nashville, TN USA; 5 Veterans Affairs Tennessee Valley Healthcare System, Nashville, TN USA

**Keywords:** Bacterial infection, Inflammation, Innate immune cells

## Abstract

The intestinal immune response is crucial in maintaining a healthy gut, but the enhanced migration of macrophages in response to pathogens is a major contributor to disease pathogenesis. Integrins are ubiquitously expressed cellular receptors that are highly involved in immune cell adhesion to endothelial cells while in the circulation and help facilitate extravasation into tissues. Here we show that specific deletion of the *Tln1* gene encoding the protein talin-1, an integrin-activating scaffold protein, from cells of the myeloid lineage using the *Lyz2*-cre driver mouse reduces epithelial damage, attenuates colitis, downregulates the expression of macrophage markers, decreases the number of differentiated colonic mucosal macrophages, and diminishes the presence of CD68-positive cells in the colonic mucosa of mice infected with the enteric pathogen *Citrobacter rodentium*. Bone marrow-derived macrophages lacking expression of *Tln1* did not exhibit a cell-autonomous phenotype; there was no impaired proinflammatory gene expression, nitric oxide production, phagocytic ability, or surface expression of CD11b, CD86, or major histocompatibility complex II in response to *C. rodentium*. Thus, we demonstrate that talin-1 plays a role in the manifestation of infectious colitis by increasing mucosal macrophages, with an effect that is independent of macrophage activation.

## Introduction

Macrophages are an essential component of the intestinal immune response. Under normal conditions, the intestinal lamina propria is home to the largest population of mononuclear leukocytes in the body with macrophages being the most abundant subpopulation^1^. Intestinal macrophages are unique compared to other tissue macrophages in that they are continuously replenished from circulating blood monocytes as they help maintain epithelial renewal and immune homeostasis in the presence of the gut microbiota^1^. Inflammatory conditions of the intestines are often the result of perturbation of the normal gut flora, pathogenic infection, or an inherent dysregulation of the immune response. During infection with pathogenic bacteria, such as enteropathogenic *Escherichia coli* (EPEC) and Shiga toxin-producing *E. coli*, the enhanced immune cell recruitment and activation can be both protective and deleterious, and is a major cause of the resulting pathology^2^.

Recruited circulating monocytes enter tissues through integrin binding to adhesion molecules on endothelial cells^3–5^. Integrin ligand affinity and activation is facilitated by talin-1, a cytoskeletal scaffold protein that interacts with the cytoplasmic domain of the β-subunit of integrins and induces a confirmational change to the extracellular domain^6,7^. In this study, we sought to elucidate the role of talin-1 in macrophages during colonic infection by *Citrobacter rodentium*, the rodent equivalent of EPEC, a well-established model of infectious colitis^8–10^. Myeloid cell-specific knockdown of *Tln1* using the *Lyz2*-cre driver mouse resulted in decreased epithelial damage and inflammation that was associated with decreased transcript levels of macrophage markers, less macrophages, and less differentiated macrophages in the infected tissues. This outcome was independent of a cell-autonomous effect of talin-1 in macrophage activation and function ex vivo. Overall, our data indicate that talin-1 regulates the recruitment of macrophages in response to the enteric murine pathogen *C. rodentium* and thus facilitates the inflammatory response.

## Results

### Mice lacking talin-1 in the myeloid cell lineage exhibit less epithelial damage and inflammation during pathogenic colitis

**Figure 1:**
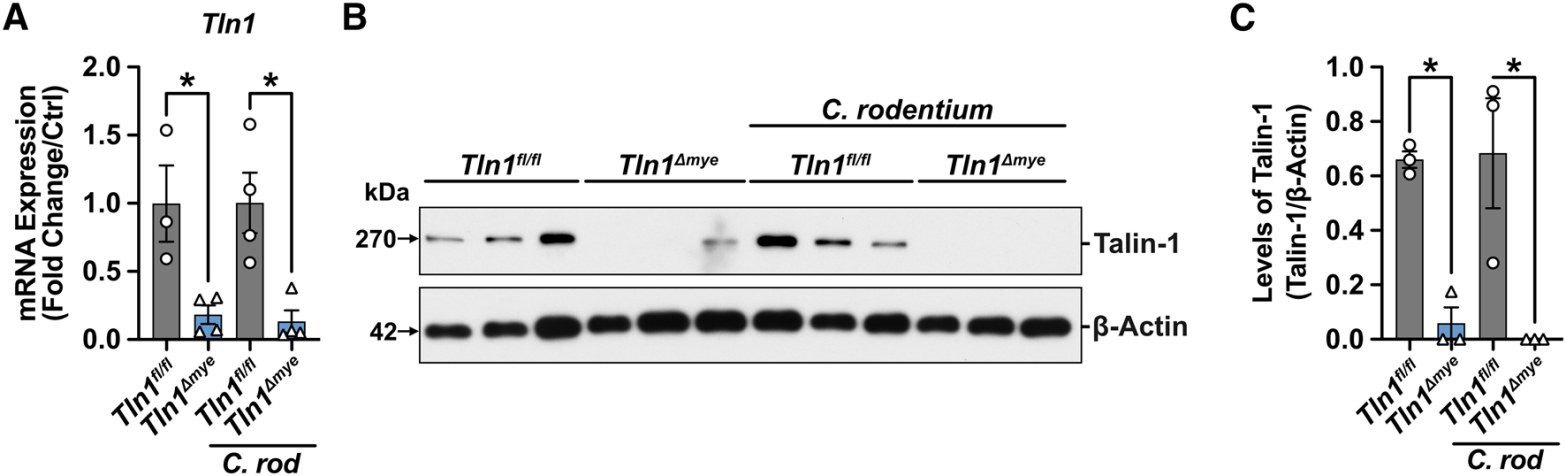
Myeloid cell-specific deletion of *Tln1*. BMmacs were generated form *Tln1*^*fl/fl*^ and *Tln1*^*Δmye*^ mice and infected with *C. rodentium*. (**A**) Expression of *Tln1* mRNA determined by RT-real-time PCR 6 h post-infection; *n* = 3–4 mice per genotype. (**B**) Western blot of talin-1 protein (270 kDa) expression and (**C**) densitometry analysis at 24 h post-infection; *n* = 3 mice per group. All values are reported as mean ± SEM. Statistical analyses, where shown; *P < 0.05 determined by 1-way ANOVA and Tukey post hoc test.

**Figure 2:**
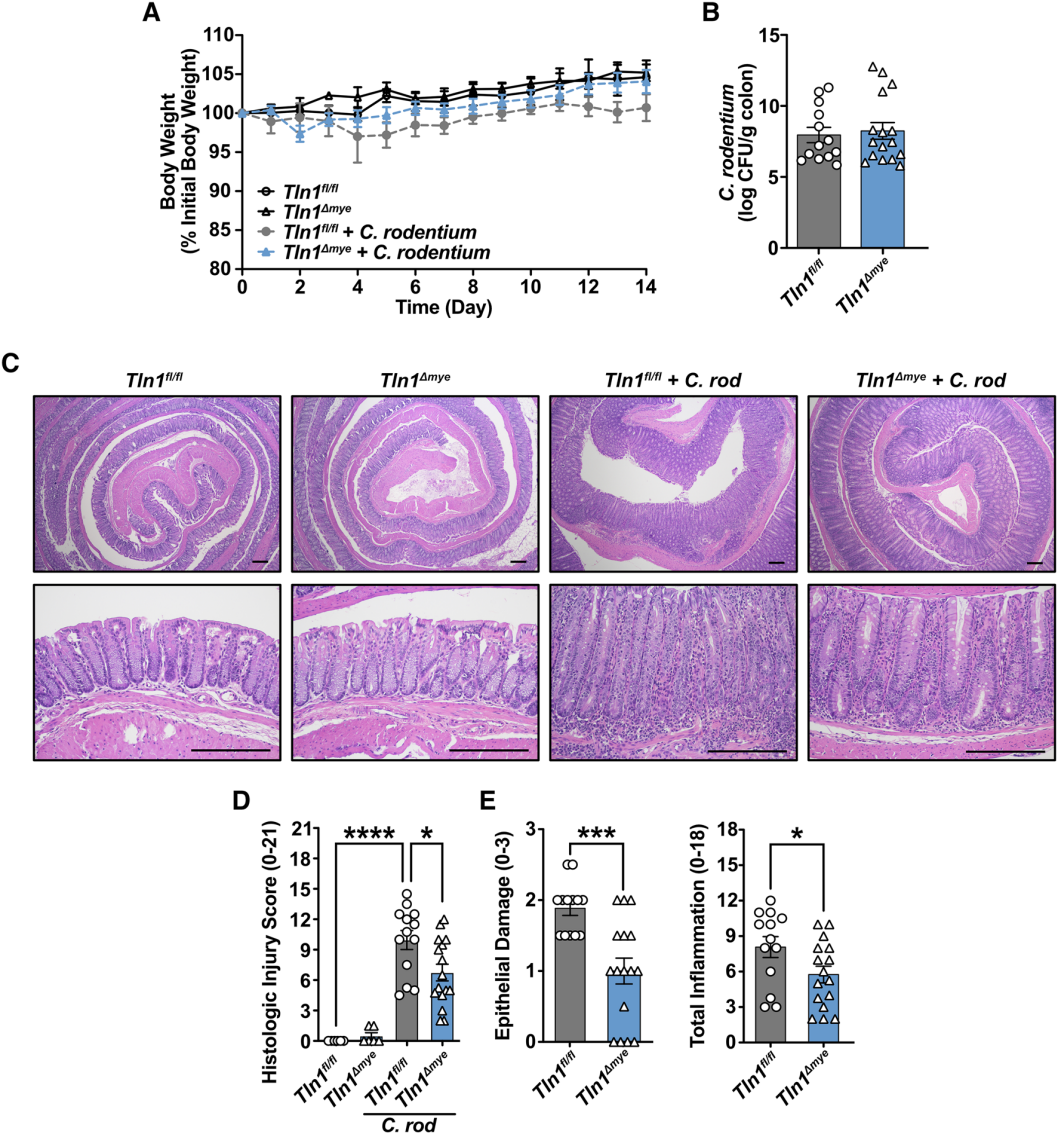
Loss of talin-1 in myeloid cells protects mice from *C. rodentium*-induced injury. *Tln1*^*fl/fl*^ and *Tln1*^*Δmye*^ male littermates were orally inoculated with 5 × 10^8^ CFU of *C. rodentium* by gavage and observed for 14 days. *n* = 7 uninfected mice and* n* = 13–16 infected mice per genotype. Data pooled from 2 independent experiments. (**A**) Daily body weights depicted as a percent of initial body weight. (**B**) *C. rodentium* colonization of the colon determined by counting CFUs from serial dilutions of homogenized tissues and normalized to tissue weight on day 14 post-infection. (**C**) Representative H&Es of Swiss-rolled fixed tissues. Note that the low and high magnification images are from the same slide for each genotype. Scale bars represent 200 μm. (**D**) Histologic injury score assessed by a pathologist using the H&E-stained tissues and is the sum of (**E**) epithelial damage and total inflammation. All values are reported as mean ± SEM. Statistical analyses, where shown; *P < 0.05, ***P < 0.001, and ****P < 0.0001 determined by (**D**) 1-way ANOVA and Tukey post hoc test and (**E**) Student’s *t* test.

*Tln1*^*fl/fl*^ mice were crossed with C57BL/6 *Lyz2*^*cre/cre*^ mice and the resulting offspring were crossed to generate *Tln1*^*fl/fl*^;*Lyz2*^+*/*+^ (*Tln1*^*fl/fl*^) and *Tln1*^*fl/fl*^;*Lyz2*^*cre/cre*^ (*Tln1*^*Δmye*^) littermates. The knockdown of *Tln1* mRNA (Fig. 1A) and talin-1 protein (Fig. 1B,C; Supplementary Fig. S1) was evidenced in bone marrow-derived macrophages (BMmacs) isolated from *Tln1*^*Δmye*^ mice and infected or not ex vivo with *C. rodentium*. Note that *C. rodentium* infection had no effect on the expression of *Tln1* mRNA (Fig. 1A) or talin-1 protein (Fig. 1B and C; Supplementary Fig. S1) in *Tln1*^*fl/fl*^ BMmacs. Male mice were then infected via oral gavage with 5 × 10^8^ colony forming units (CFUs) of *C. rodentium* and monitored for 14 days, as previously described^8–10^. We did not observe differences in body weight loss (Fig. 2A) or bacterial burden (Fig. 2B) between *Tln1*^*fl/fl*^ and *Tln1*^*Δmye*^ mice. Consistent with previous studies^11,12^, infection with *C. rodentium* resulted in mucosal hyperplasia, erosion and ulceration of the epithelium, and immune cell invasion of the mucosa and submucosa (Fig. 2C). Notably, histologic injury scores were attenuated in *Tln1*^*Δmye*^ mice compared to *Tln1*^*fl/fl*^ littermates (Fig. 2D), due to a reduction in both components of the composite score, epithelial damage, and total inflammation (Fig. 2E).

To determine if these findings are specific to bacterially-induced colitis, we treated *Tln1*^*fl/fl*^ and *Tln1*^*Δmye*^ littermates with 4% dextran sulfate sodium (DSS), a chemically-induced model of epithelial injury^9,13^. When compared to *Tln1*^*fl/fl*^ mice, *Tln1*^*Δmye*^ mice did not show differences in body weight loss (Supplementary Fig. S2A), colon length (Supplementary Fig. S2B), or histologic injury score (Supplementary Fig. S2C). These results suggest that talin-1 in myeloid cells contributes to *C. rodentium* pathogenesis and is specific to pathogenic infection.

### Mice with myeloid cell-specific deletion of *Tln1* express lower levels of macrophage markers and exhibit less macrophages in the colon with *C. rodentium* infection

**Figure 3:**
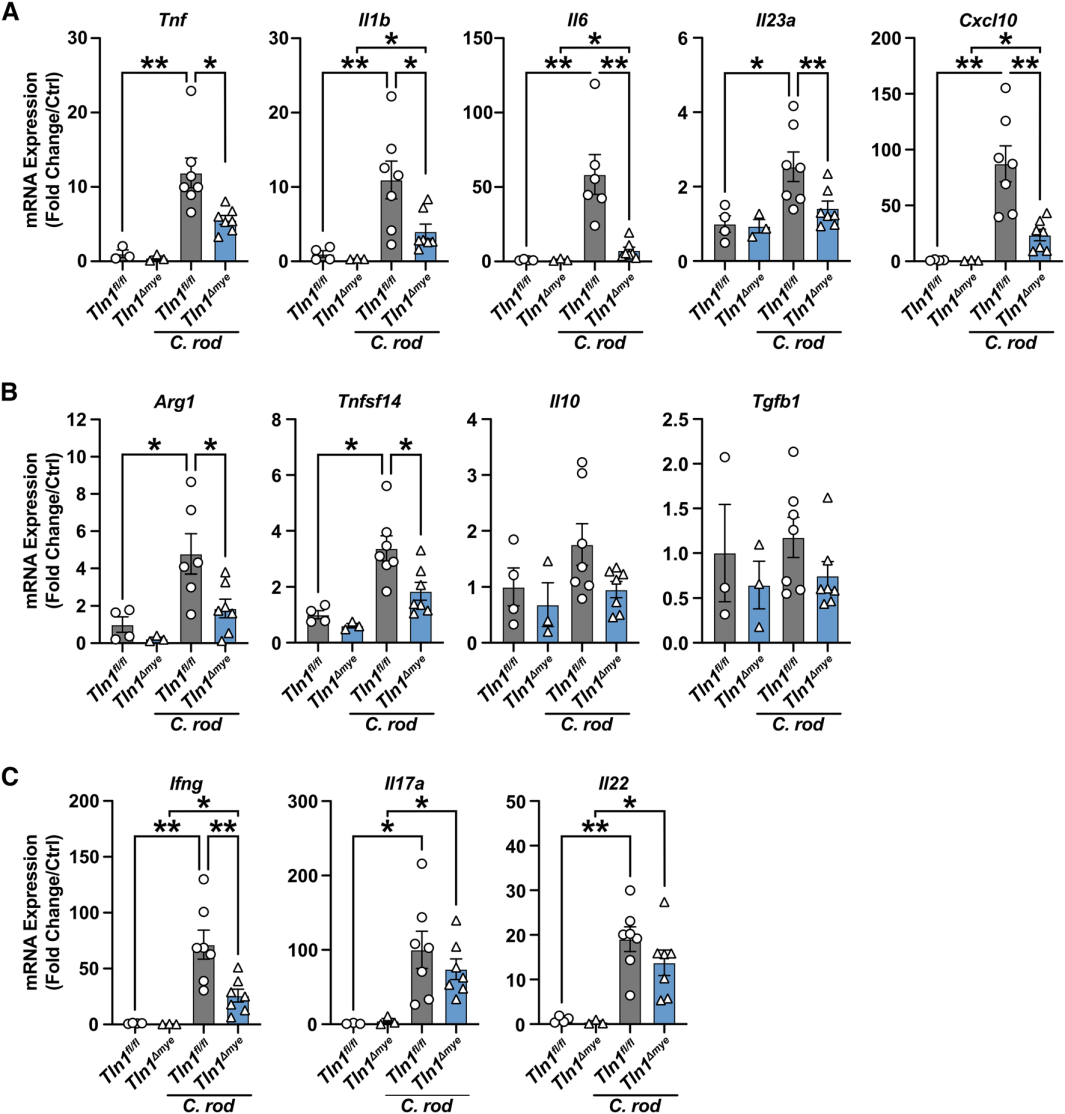
Knockdown of talin-1 in myeloid cells reduces expression of macrophage markers in vivo. Whole colon tissue mRNA expression of (**A**) proinflammatory, (**B**) anti-inflammatory, and (**C**) T cell markers analyzed by RT-real time PCR. *n* = 3–4 uninfected mice and *n* = 6–7 infected mice per genotype. Each symbol is a different mouse. All values are reported as mean ± SEM. Statistical analyses, where shown; *P < 0.05 and **P < 0.01 determined by 1-way ANOVA with Tukey post hoc test or Kruskal–Wallis test, followed by Mann–Whitney *U* tests.

We next assessed the expression of immune effectors typically associated with *C. rodentium* infection in the colon. The genes encoding for markers of inflammation, namely *Tnf*, *Il1b*, *Il6*, *Il23a*, and *Cxcl10* were induced with infection in *Tln1*^*fl/fl*^ mice and significantly decreased in the colon tissues from infected *Tln1*^*Δmye*^ mice compared to infected *Tln1*^*fl/fl*^ mice (Fig. 3A). Additionally, the levels of the transcripts encoding arginase 1 (*Arg1*) and tumor necrosis factor super family 14 (*Tnfsf14; Light*), which are anti-inflammatory during colitis (22,23), were also significantly decreased in infected *Tln1*^*Δmye*^ colon tissues (Fig. 3B). The expression levels of *Il10* and *Tgfb1* were induced in infected mice and not different between genotypes (Fig. 3B). We observed a significant reduction in the expression of the Th1 T cell marker *Ifng*, but no change in the expression of the Th17 marker *Il17a*, or the Th22 marker *Il22*, in infected *Tln1*^*Δmye*^ colon tissues (Fig. 3C).

**Figure 4:**
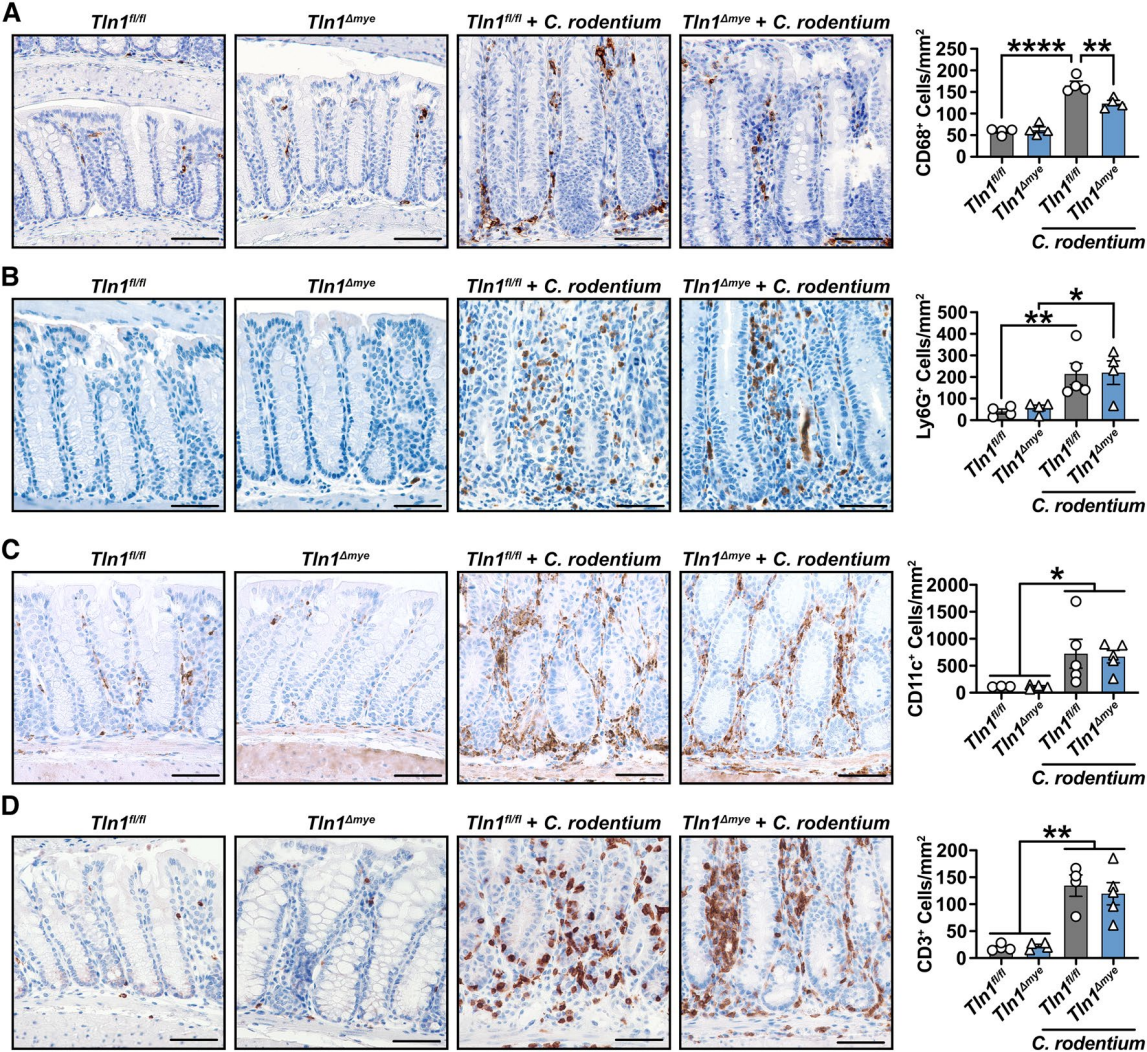
Myeloid cell talin-1 contributes to macrophage recruitment in response to infection in the mouse colon. Representative immunohistochemistry images and the quantification of positive cells per mm^2^ of Swiss-rolled colon tissues immunoperoxidase-stained for (**A**) CD68, (**B**) Ly6G, (**C**) CD11c, and (**D**) CD3. *n* = 4 uninfected mice and *n* = 5 infected mice per genotype. All values are reported as mean ± SEM. Statistical analyses, where shown; *P < 0.05, **P < 0.01, and ****P < 0.0001 determined by 1-way ANOVA and Tukey post hoc test. Scale bars represent 100 μm.

Thus, we next assessed the population of immune cells present in the colon via immunohistochemistry and quantification of positively-stained cells. Consistent with the gene expression we observed, the CD68^+^ macrophages in the colon were increased with infection in *Tln1*^*fl/fl*^ tissues, but were significantly less abundant in infected *Tln1*^*Δmye*^ mice (Fig. 4A). As expected during infection, there were increases in the number of Ly6G^+^ neutrophils and CD11c^+^ dendritic cells, but there were no differences between *Tln1*^*fl/fl*^ and *Tln1*^*Δmye*^ mice (Fig. 4B,C). The *Lyz2*-flox system is used to target specific gene deletions in cells of the myeloid cell lineage and affects macrophages, neutrophils, and dendritic cells to varying degrees^14^ suggesting that talin-1 is important for the recruitment of macrophages, but not dendritic cells or neutrophils during *C. rodentium* infection. In addition, the number of T cells, marked by CD3^+^, were similar in *Tln1*^*Δmye*^ mice compared to *Tln1*^*fl/fl*^ mice with infection (Fig. 4D), matching the comparable expression of *Il17a* and *Il22*.

### The number of differentiated macrophages in the gut is decreased in *Tln1*^*Δmye*^ mice

**Figure 5:**
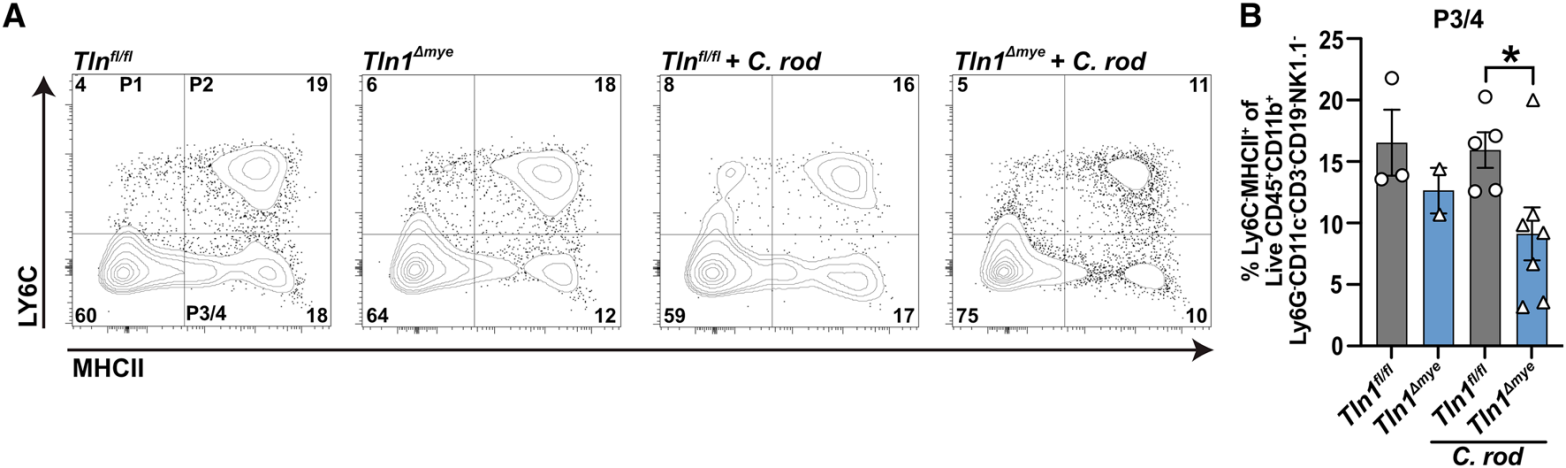
*Tln1*^*fl/fl*^ mice exhibit increased macrophage differentiation in the infected colon compared to *Tln1*^*Δmye*^ mice. Cells isolated from the colonic lamina propria of *Tln1*^*fl/fl*^ and *Tln1*^*Δmye*^ mice infected with *C. rodentium* or mock, were assessed by flow cytometry for P1, P2, and P3/4 differentiation makers (**A**). Data shown are the concatenated results from all mice in the experiment. The frequency of Ly6C^–^MHCII^+^ macrophages (P3/4) is depicted in (**B**). *n* = 2–3 uninfected mice and *n* = 5–7 infected mice per genotype. All values are reported as mean ± SEM. Statistical analyses, *P < 0.05 determined by 1-way ANOVA with a Kruskal–Wallis test, followed by Mann–Whitney *U* tests.

The reduced expression of macrophage markers in the colon of infected of *Tln1*^*Δmye*^ mice (see Figs. 3 and 4) suggests that less macrophages were present in the mucosa. To test this postulate, we isolated immune cells from the whole colon of uninfected and infected *Tln1*^*fl/fl*^ and *Tln1*^*Δmye*^ mice and assessed monocyte-macrophages by flow cytometry using previously defined parameters^15^. We identified three subsets of CD45^+^CD11b^+^ macrophages based on their expression of Ly6C and MHCII^15^. The waterfall phenotype, which is a feature of macrophage differentiation in the mucosa, was observed in the uninfected and infected *Tln1*^*fl/fl*^ and *Tln1*^*Δmye*^ mice (Fig. 5A). While monocyte Population 1 (P1; Ly6C^+^MHCII^–^ cells) and Population 2 (P2; Ly6C^+^MHCII^+^ cells) were not significantly different between the infected groups (Fig. 5A; Supplementary Fig. S3), there was a significant decrease in Population 3/4 (P3/4; Ly6C^–^MHCII^+^ cells), which correspond to differentiated macrophages, in the infected *Tln1*^*Δmye*^ mice compared to the infected *Tln1*^*fl/fl*^ mice (Fig. 5A,B).

### Loss of talin-1 does not have a cell-intrinsic effect on macrophage activation and function ex vivo

**Figure 6:**
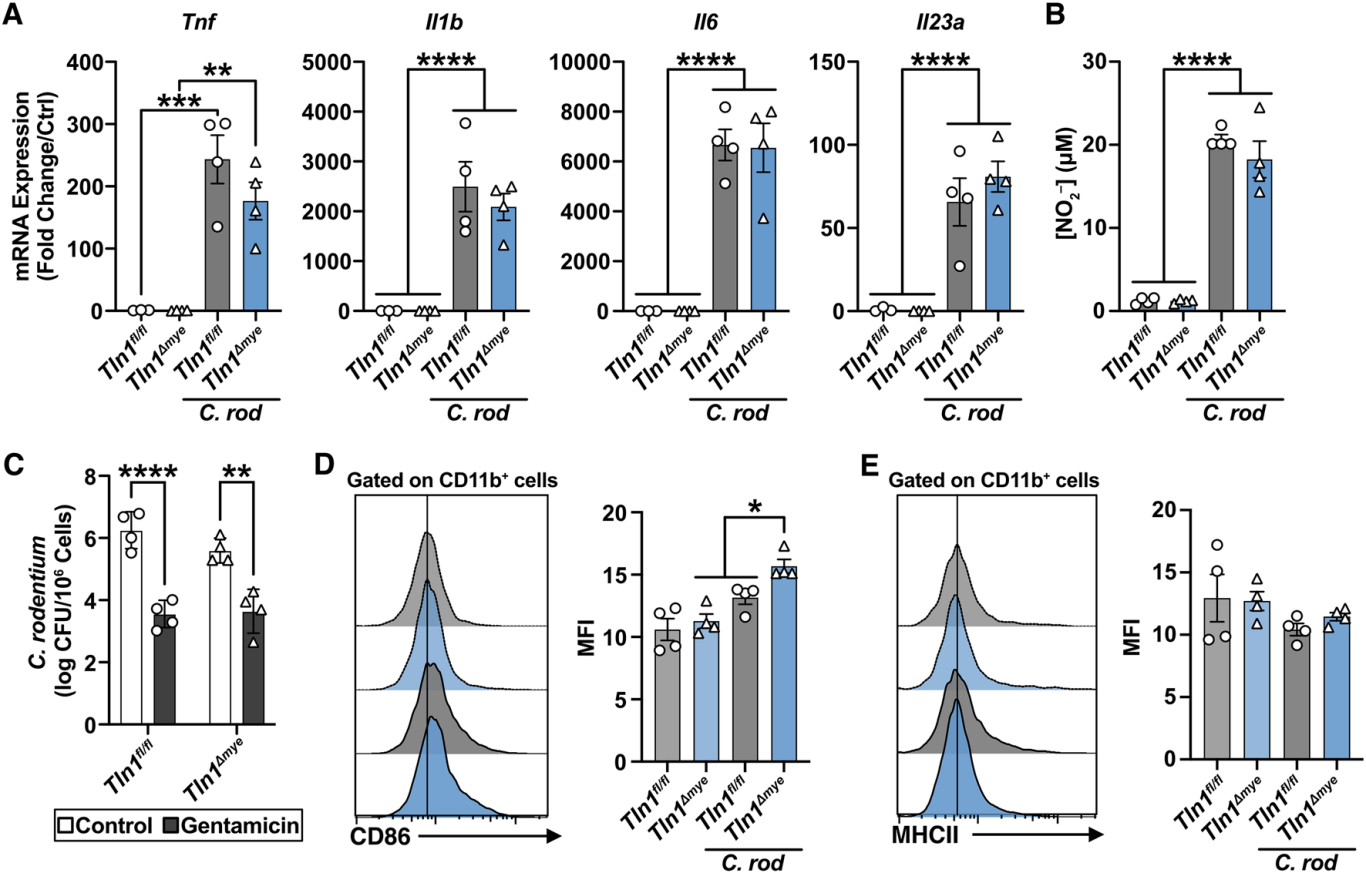
Talin-1 does not contribute to macrophage activation or phagocytic ability. BMmacs derived from *Tln1*^*fl/fl*^ and *Tln1*^*Δmye*^ mice were infected or not with *C. rodentium*, *n* = 3–4 mice per genotype. (**A**) Expression of pro-inflammatory macrophage markers at 6 h post-infection. (**B**) The concentration of NO_2_^–^ in cell supernatants 24 h post-infection measured by the Griess reaction. (**C**) The amount of *C. rodentium* phagocytosed by *Tln1*^*fl/fl*^ and *Tln1*^*Δmye*^ BMmacs 1 h post-infection determined by gentamicin assay. (**D-E**) Representative flow plots and graphs depicting the surface expression and mean fluorescence intensity (MFI) of (**D**) CD86 and (**E**) MHCII on BMmacs 24 h post-infection. All values are reported as mean ± SEM. Statistical analyses, where shown; **P < 0.01, ***P < 0.001, and ****P < 0.0001 determined by 1-way ANOVA and Tukey post hoc test.

We also tested whether talin-1 has a direct role in macrophage activation and function, independent of the colonic mucosal niche. To study talin-1 in macrophages ex vivo, we generated bone marrow-derived macrophages (BMmacs) and confirmed CD11b expression, a β_2_ integrin that is a marker of macrophages. *Tln1*-deficient BMmacs expressed a modest 6% increase of CD11b compared to *Tln1*^*fl/fl*^ BMmacs with and without infection (Supplementary Fig. S4). This indicates that loss of talin-1 did not impede macrophage differentiation ex vivo. BMmacs derived from *Tln1*^*fl/fl*^ and *Tln1*^*Δmye*^ mice displayed similar levels of induced *Tnf, Il1b*, *Il6*, and *Il23a* transcripts after infection with *C. rodentium* (Fig. 6A). In addition, *Tln1*-deficient BMmacs retained the ability to produce nitric oxide (Fig. 6B), a commonly used marker to evaluate the activation and polarization of inflammatory macrophages^16,17^. We also assessed the effect of *Tln1* deletion in bone marrow-derived dendritic cells (BMDCs; Supplementary Fig. S5A). We found that stimulation with *C. rodentium* similarly did not affect gene expression of *Tnf*, *Il1b*, *Il6*, or *IL23a* (Supplementary Fig. S5B), or levels of nitric oxide (Supplementary Fig. S5C). These data suggest that the decreased gene expression and inflammation that we observed in the tissues of *Tln1*^*Δmye*^ mice during *C. rodentium* infection is not due to impaired capacity for macrophage or dendritic cell activation.

To further assess the role of talin-1 in the function of macrophages, we accessed phagocytosis and antigen presentation. We found that *Tln1*-deficient BMmacs engulfed the same number of *C. rodentium* as *Tln1*^*fl/fl*^ BMmacs (Fig. 6C), signifying that talin-1 is not required for the phagocytosis of *C. rodentium* by macrophages. We next determined if talin-1 might have a role in the ability of macrophages to present antigens. We therefore measured the surface expression of MHCII and the co-stimulatory molecule, CD86. While the percentage of CD11b^+^ cells were not different between genotypes, we found that CD11b^+^ *Tln1*-deficient BMmacs expressed higher levels of CD86 (Fig. 6D; Supplementary Fig. S6), but not MHCII (Fig. 6E; Supplementary Fig. S6), compared to *Tln1*^*fl/fl*^ BMmacs with infection. These data suggest that talin-1 is not required for the expression of markers of antigen-presenting cells.

## Discussion

Talin-1 is a large cytoplasmic protein that creates a mechanical link between the actin cytoskeleton and integrins. Previous studies have demonstrated that talin-1 is required for the migration and recruitment of leukocytes, including neutrophils, dendritic cells, and T cells to sites of inflammation^18–20^. In general, migration of monocytes into tissues has been linked to the involvement of integrins. Specific to our findings, talin-1 has been directly linked to monocyte adhesive events ^21,22^. Using mice with myeloid-specific knockdown of *Tln1*, we demonstrate that talin-1 is essential in regulating the number of macrophages in the colonic mucosa during *C. rodentium* infection. Talin-1 did not have a cell-intrinsic role in macrophages as the loss of *Tln1* did not affect gene expression, NO production, or phagocytosis of *C. rodentium *ex vivo. Therefore, considering the prior literature^3–5,21,23^ and our results, we conclude that the most likely explanation for the reduced inflammation that we observe is due to impaired tissue migration of monocytes, which develop into macrophages in the colonic mucosa. Collectively, our data demonstrate that loss of macrophage talin-1 is protective in the *C. rodentium* model of infectious colitis through a non-cell-autonomous manner and suggest that talin-1 is a promising target to limit the recruitment of macrophages during infectious colitis.

When infected with the mouse pathogen *C. rodentium*, mice that lacked expression of *Tln1* in myeloid cells exhibited lower histologic injury scores and a decrease in both proinflammatory and anti-inflammatory gene expression in the colonic tissue. The decrease in histologic injury was a result of decreases in both epithelial injury and inflammation, but there was no change in the level of *C. rodentium* burden in the colon. It has been previously shown that mice with a weakened response of neutrophils and/or macrophages, but have an intact lymphoid cell compartment, exhibit decreased colitis and epithelial damage, yet can still control bacterial growth^24,25^. In contrast, immunocompromised mice or mice that lack an effective type 3 T cell or innate lymphoid cell response are unable to control the bacterial burden and exhibit higher rates of mortality^26–28^.

We observed that *Tln1*^*Δmye*^ mice displayed decreased numbers of CD68^+^ cells in the mucosa while maintaining similar numbers of neutrophils, dendritic cells, and T cells as marked by Ly6G, CD11c, and CD3, respectively, to their genetically normal littermates. Furthermore, the monocyte-macrophage waterfall assay showed less differentiated macrophages in the infected *Tln1*^*Δmye*^ mice compared to *Tln1*^*fl/fl*^ animals, suggesting that fewer monocytes were infiltrating into the lamina propria at the beginning of the infection. This reduction in the number of macrophages in the colon of *Tln1*^*Δmye*^ mice could be due to different events, including lower recruitment to the site of infection or migration through the endothelium to the lamina propria, or increased cell death in the mucosa. In addition, the decrease in transcript expression that we observed in *Tln1*^*Δmye*^ mice are from genes that are normally associated with proinflammatory and anti-inflammatory cells of the innate immune response. A decrease in proinflammatory cytokines creates a feedback loop that then results in less macrophage activation, and less inflammation and tissue damage. These data suggest that the decrease of expression of these genes may be due to the decreased number of macrophages and not due to polarization state. Supporting this concept, we found that BMmacs and BMDCs from *Tln1*^*fl/fl*^ and *Tln1*^*Δmye*^ mice are similarly activated ex vivo by *C. rodentium*. It should also be noted that some heterogeneity in cell populations can be observed after ex vivo stimulation, such as the presence of macrophages in GM-CSF-treated bone marrow cells^29^. Our data suggest that talin-1 is not involved in the process of macrophage stimulation. However, further experiments, including transfer of macrophages from *Tln1*^*fl/fl*^ and *Tln1*^*Δmye*^ mice into *Ccr2*-deficient animals, which are characterized by an empty intestinal macrophage niche^30^, or by mixed bone marrow chimera experiments with cells from both genotypes, could be utilized to support the conclusion that talin-1 plays a role in the recruitment of macrophages in the intestine. We demonstrate no differences in the mRNA levels of *Il22* and *Il17* which are often expressed by Th22 and Th17 cells in the colon, respectively, and are important for controlling bacterial growth, but not disease development during enteropathogenic infection^27,31,32^.

Integrins are a vital component of innate leukocytes and are involved in bidirectional signaling across the membrane. We therefore were interested in whether the decrease in colitis results from decreased macrophage function. Talin-1 has also been implicated in the phagocytic ability of macrophages; however, this is dependent on the integrin β-subunit and the target of the engulfment. Talin-1 is dispensable for integrin β_5_-mediated phagocytosis of apoptotic cells^33^, while talin-1 is required for the integrin β_2_-mediated phagocytosis of C3bi-opsinized red blood cells^34^. We observed that talin-1 was not necessary for the phagocytosis of *C. rodentium* by macrophages. These cells also have the dual responsibility of directly killing pathogens and acting as professional antigen presenting cells to activate and direct T cells^35^. Talin-1 has been shown to be involved in T cell activation in an antigen presenting cell contact-dependent manner through stabilization of the immune synapse^36^. Lim et al. demonstrate that *Tln1*-deficient dendritic cells using the *Cd11c-*cre expressed lower levels of CD86 and MHCII when stimulated with LPS that was also associated with decreased gene expression of proinflammatory markers^20^. However, we show using a different Cre driver and stimuli that there were no differences in induced expression of proinflammatory markers in response to *C. rodentium* by dendritic cells or macrophages. We did not observe any differences in the ability of *Tln1*-deficient macrophages to express MHCII but show that *Tln1*-deficient macrophages express modestly higher surface levels of CD86 and the β_2_ integrin CD11b, which can be used to identify inflammatory macrophages in the lung during acute inflammation^37^. In addition, myeloid-derived talin-1 does not contribute to DSS colitis. These data indicate that talin-1 is not consistently essential for macrophage or dendritic cell differentiation or function, but our other data show that talin-1 is important for the presence of differentiated macrophages in the colonic mucosa during challenge with pathogenic bacteria.

In summary, the results from this study indicate that myeloid talin-1 affects the number of differentiated macrophages in the colon in the context of pathogenic colitis. Our data indicate that the expression of talin-1 by macrophages contributes to *C. rodentium*-induced colonic inflammation, with the increased number of colonic macrophages leading to more generation of inflammatory mediators, while failing to help eradicate the pathogen. These findings provide further insight into the development of bacteria-induced colitis and highlights a potential target to control macrophage movement into tissues that leads to the propagation of inflammation.

## Materials and methods

### Mice

C57BL/6 *Tln1*^*fl/fl*^ mice provided by Dr. Roy Zent at Vanderbilt University Medical Center (Nashville, TN). These mice were crossed with C57BL/6 *Lyz2*^*cre/cre*^ mice and the resulting offspring were crossed to generate *Tln1*^*fl/fl*^;*Lyz2*^+*/*+^ (*Tln1*^*fl/fl*^) and *Tln1*^*fl/fl*^;*Lyz2*^*cre/cre*^ (*Tln1*^*Δmye*^) littermates. Animals were euthanized by exposure to carbon dioxide followed by cervical dislocation. All experiments were approved by the IACUC at Vanderbilt University and Institutional Biosafety Committee and the Research and Development Committee of the Veterans Affairs Tennessee Valley Healthcare System under the protocol V2000018 and performed in accordance with these institutional guidelines. The study was carried out in compliance with the ARRIVE guidelines.

### Generation of bone marrow-derived immune cells

BMmacs and BMDCs were generated as described^17^. Briefly, cells were flushed from the femur and tibia of naïve mice, and RBCs were lysed prior to counting and plating using ACK Lysing Buffer (Thermo Fisher). Cells were differentiated for 7 days in complete medium (DMEM media supplemented with 10% FBS, 100 U/ml penicillin/streptomycin, 25 mM HEPES, and 20 ng/ml recombinant M-CSF (PeproTech). BMDCs were differentiated for 8 days in complete medium (DMEM media supplemented with 10% FBS, 100 U/ml penicillin/streptomycin, 25 mM HEPES, and 10 ng/ml recombinant GM-CSF (PeproTech).

### Infection with* C. rodentium*

Male adult (6–12 wk) littermates were orally inoculated by gavage with a sublethal dose, 5 × 10^8^, *C. rodentium* strain DBS100 as previously described^8–10^. Control mice received sterile Luria–Bertani broth alone. Animals were monitored and weighed daily for 14 days. Mice were sacrificed, and the colons were removed, measured, cleaned, weighed, and Swiss-rolled for fixation in 10% neutral buffered formalin for histology. Prior to fixation, three proximal and distal pieces were collected; two pieces were flash frozen and the third was weighed, homogenized, serially diluted, and cultured on McConkey agar plates to determine bacterial colonization by counting the CFUs. Hematoxylin and eosin (H&E) stained slides were scored by a gastrointestinal pathologist (M.B.P.), blinded to the mouse genotype and infection status, for histologic injury (0–21), a composite of the total inflammation (0–18) plus epithelial injury (0–3) with 0: no injury; 0.5: mucus depletion 1: erosion (mild to moderate); 2: superficial ulcers or extensive erosion; 3: deep ulcers and/or necrosis of the mucosa^8–10^. Total inflammation is the combination of acute inflammation (0–3) based on polymorphonuclear leukocytes, plus chronic inflammation (0–3) based on mononuclear cell infiltrates and multiplied by the depth of inflammation (0–3) with 0: no inflammation; 1: mucosa; 2: submucosa; 3: muscular propria or beyond.

BMmacs and BMDCs were infected with *C. rodentium* at a multiplicity of infection of 10 for 3 h in antibiotic-free media. The cells were then washed and provided media containing penicillin and streptomycin for an additional 3 h (mRNA) or 21 h (protein, flow cytometry).

### DSS-induced model of colitis

Male adult littermates were treated with 4% DSS in the drinking water for 5 days after which the DSS was removed, and the mice remained on regular water for an additional 5 days prior to euthanasia^9,13^. Mice were sacrificed, and the colons were removed, measured, cleaned, weighed, and Swiss-rolled for fixation in 10% neutral buffered formalin for histology. Hematoxylin and eosin (H&E) stained slides were scored by a gastrointestinal pathologist (M.K.W.), blinded to the mouse genotype and treatment status, for histologic injury (0–40), a composite of the total inflammation (0–24) plus crypt damage (0–16). Total inflammation is a combination of inflammation (0–3) plus depth of inflammation (0–3) with 0: no inflammation; 1: mucosa; 2: submucosa; 3: transmural multiplied but the percent of involved area (1–4) with 1: ≤ 25%; 2: 26–50%; 3: 51–75%; 4: > 75%. Crypt damage is a combination of the amount of damage (1–4) with 1: basal 1/3 damaged; 2: basal 2/3 damaged; 3: only surface intact; 4: entire crypt and surface lost plus the percent of the tissue involved by crypt damage with 1: 10–25%; 2: 26–50%; 3:56–75%; 4: > 75%.

### mRNA analysis

Total RNA was isolated from cells and the flash frozen colonic tissues using the RNeasy Mini Kit (QIAGEN). Total RNA samples were reverse transcribed into cDNA using the SuperScript III Reverse Transcriptase (Thermo Fisher), Oligo (dT) primers (Thermo Fisher), and dNTP Mix (Applied Biosystems). Real-time PCR was performed using the PowerUp SYBR Green Master Mix (Applied Biosystems). The primers are listed in Supplementary Table S1.

### Western blot and densitometric analysis

Protein isolation, SDS-PAGE separation, nitrocellulose membrane transfer, and band visualization were performed as described^17^. The membranes were incubated with a rabbit anti-talin-1 (Cell Signaling), or a mouse anti-β-actin (Sigma) followed by HRP-labeled goat anti-rabbit IgG (Jackson ImmunoResearch) or HRP-labeled goat anti-mouse IgG (Promega), respectively. Densitometric analysis was performed with Fiji (ImageJ)^38^.

### Immunohistochemistry

Paraffin-embedded Swiss-rolled murine colon tissues were processed as previously described^17^ overnight at 4 °C using the following antibodies: anti-CD68 (pre-diluted, Biocare Medical), anti-Ly6G (1:1000; Abcam), anti-CD11c (Cell Signaling), or anti-CD3 (1:150; Abcam). All slides were imaged and analyzed using a Cytation C10 Confocal Imaging Reader and Gen 5+ software (Agilent BioTek). The average number of CD68-, Ly6G-, Cd11c-, and CD3-positive cells was quantified by the Cell Analysis function of the Gen 5+ software and was limited to the mucosa to reduce inclusion of non-specific staining and normalized to tissue surface area.

### Measurement of NO_2_^–^

NO_2_^−^ concentrations were assessed using the standard Griess reaction (Promega) at 24 h post-infection with *C. rodentium*.

### Gentamicin assay

BMmacs were infected at a multiplicity of infection of 10 for 1 h prior to washing, counting, and treating with 200 g/ml gentamicin for 1 h^39^. The cells were then lysed with 0.1% saponin for 30 min at 37 °C and the lysate was serially diluted and cultured on McConkey agar plates. CFUs were counted and normalized to cell count.

### Colonic lamina propria isolation

Isolation of the colonic lamina propria was performed as previously described^40^. Briefly, colons were removed from the mice, cut longitudinally, washed with cold PBS, cut into 5 mm pieces, and incubated in 25 ml of pre-warmed RPMI 1640 media with 5% heat-inactivated FBS, 5 mM EDTA, 1 mM dithiothreitol (Thermo Fisher Scientific), and 25 mM HEPES at 37 °C for 40 min in a non-CO2 MaxQ4450 horizontal shaker (Thermo Fisher Scientific). The media was strained through a sieve (Everyday Living) and the intestinal pieces were placed into 25 ml cold RMPI media with 2 mM EDTA and 25 mM HEPES. The tubes containing the tissues were shaken 20 times and strained. The intestinal pieces were minced and placed into 25 ml pre-warmed RPMI 1640 media containing 0.1 mg/ml Liberase TL (Roche), 0.05% DNAse I (Sigma-Aldrich), and 25 mM HEPES and shaken at 37 °C for 30 min. The cell/media mixture was pulled through a 10 ml syringe 20 times and filtered through a 70 μm cell strainer into an equal volume of cold RPMI 1640 media containing 5% heat-inactivated FBS, 0.05% DNAse I, 25 mM HEPES on ice. Cells were spun for 10 min at 4 °C and 475 × *g* and resuspended in 40% Percoll (Sigma-Aldrich) solution and underlaid using 90% Percoll. The 40/90 gradient was spun for 25 min at 20 °C at 475 × *g* with no brake or acceleration applied, resulting in an interphase layer. The interphase layer was recovered using a 1 ml pipet and then washed in fluorescence activated cell sorting (FACS) buffer (PBS with 2% FBS and 2 mm EDTA) and spun again for 10 min at 20 °C and 475 × *g* before staining. The yield of lamina propria cells per colon was 738,612 ± 189,455 (*n* = 5) in uninfected mice and 267,929 ± 75,895 (*n* = 12) in *C. rodentium*-infected mice.

### Monocyte-macrophage waterfall assay

Cells isolated from the colonic lamina propria were blocked with TruStain FcXtm (Biolegend) for 5 min at 4 °C in the dark and then incubated in the antibody cocktail (Supplementary Table S2) in PBS for 15 min at 4 °C in the dark. Cells were gated on live single CD45^+^CD11b^+^Lineage^neg^. Lineage markers used were CD11c, CD3, CD19, NK1.1, and Ly6G. A live/dead stain (Thermo Fisher Scientific) was also used to restrict analysis to live cells. Data were acquired using a Cytek® Aurora CS and flow cytometric analysis was performed using FlowJo (BD Biosciences).

### Immunophenotyping by flow cytometry

BMmacs infected or not with *C. rodentium* for 24 h were washed with PBS and fixed with CytoFix (BD Biosciences) for 20 min at 4 °C, washed, and then labeled with anti-CD11b (1:200; BD Biosciences), anti-CD86 (1:200; Invitrogen), and anti-MHCII (1:200; Invitrogen).

### Statistics

All the data shown represent the mean ± SEM. *C. rodentium* infection data is pooled from two independent experiments. A minimum of three biological replicates were used for ex vivo studies. GraphPad Prism 9.4 (GraphPad Software) was used to perform statistical analyses and significance was set at *P* < 0.05. For normally distributed data, a 2-tailed Student’s *t* test or a 1-way ANOVA with the Tukey post hoc test were performed to compare differences between two or more test groups, respectively. Non-normally distributed data was analyzed by a 1-way ANOVA with the Kruskal–Wallis test followed by a Mann–Whitney *U* test, or square root transformed and reassessed.

### Supplementary Information

Supplementary Information.

## Data Availability

The data of this study are available from the corresponding author upon reasonable request.
