# Supplementary material for: Myeloid deletion of talin-1 reduces mucosal macrophages and protects mice from colonic inflammation
Source: Sci Rep. 2023 Dec 15;13:22368. doi: 10.1038/s41598-023-49614-z (PMC10724268; doi:10.1038/s41598-023-49614-z)
Supplement: Supplementary file 1 — Supplementary Information. [file 41598_2023_49614_MOESM1_ESM.pdf]

SUPPLEMENTARY INFORMATION:

Myeloid deletion of talin-1 reduces mucosal macrophages and protects mice from colonic inflammation

Yvonne L. Latour, Kara M. McNamara, Margaret M. Allaman, Daniel P. Barry, Thaddeus M. Smith, Mohammad Asim, Kamery J. Williams, Caroline V. Hawkins, Justin Jacobse, Jeremy A. Goettel, Alberto G. Delgado, M. Blanca Piazuelo, M. Kay Washington, Alain P. Gobert & Keith T. Wilson

Supplementary Fig. S1

Supplementary Fig. S2

Supplementary Fig. S3

Supplementary Fig. S4

Supplementary Fig. S5

Supplementary Fig. S6

Supplementary Table S1

Supplementary Table S2

Talin-1

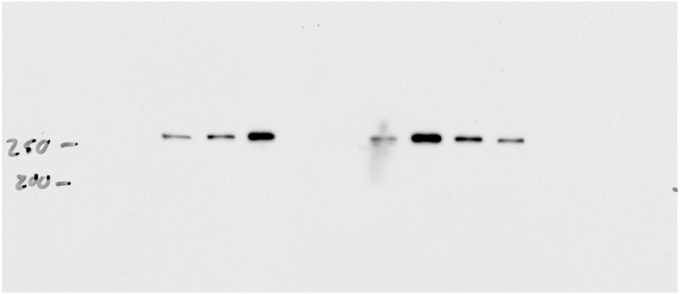

β-actin

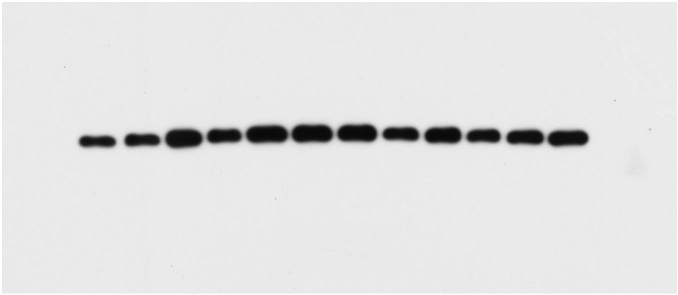

Figure S1. Uncropped images corresponding to Fig. 1B.

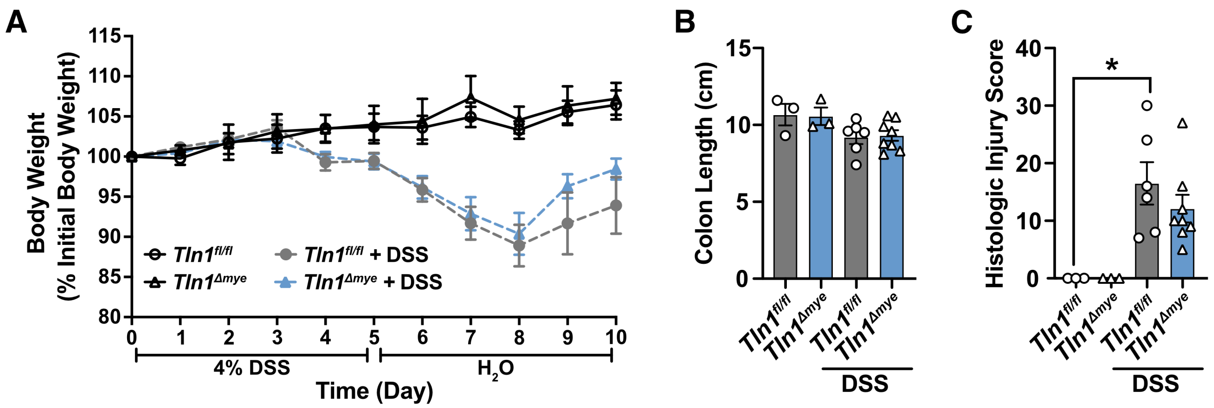

Figure S2. Loss of talin-1 in myeloid cells does not affect 4% DSS-induced injury. *Tln1$^{fl/fl}$* and *Tln1$^{\Delta mye}$* male littermates were treated with 4% DSS in the drinking water for 5 days followed by 5 days recovery on regular water. *n* = 3 uninfected mice and *n* = 7-8 infected mice per genotype. Data pooled from 2 independent experiments. (**A**) Daily body weights depicted as a percent of initial body weight. (**B**) Colon length. (**C**) Histologic injury score assessed by a pathologist. All values are reported as mean ± SEM. **P*<0.05 determined by 1-way ANOVA and Tukey post hoc test.

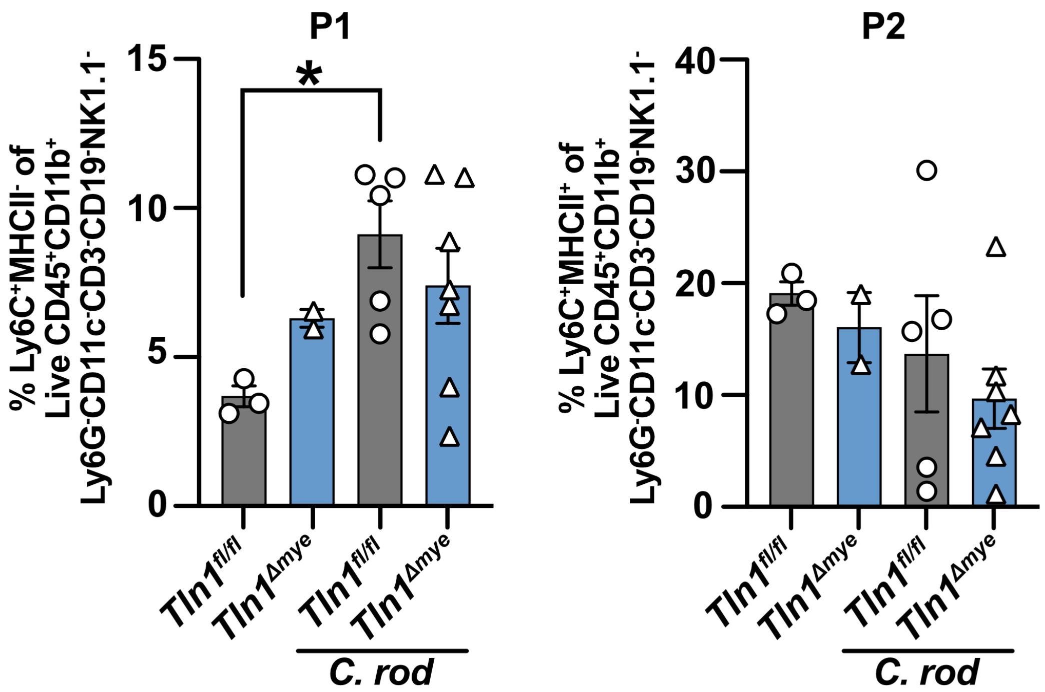

Figure S3. Recruitment of monocytes in the colonic mucosa. *Tln1*$^{fl/fl}$ and *Tln1*$^{\Delta mye}$ mice were infected or not with *C. rodentium* for 14 days. Cells from the lamina propria were isolated and the monocyte-macrophage waterfall assay was performed. The percent of P1 (Ly6C^{+}MHCII^{-} cells) and P2 (Ly6C^{+}MHCII^{+} cells) populations are shown. n = 2-3 uninfected mice and n = 5-7 infected mice per genotype. All values are reported as mean ± SEM. Statistical analyses, *P<0.05 determined by 1-way ANOVA with a Kruskal-Wallis test, followed by Mann-Whitney U tests.

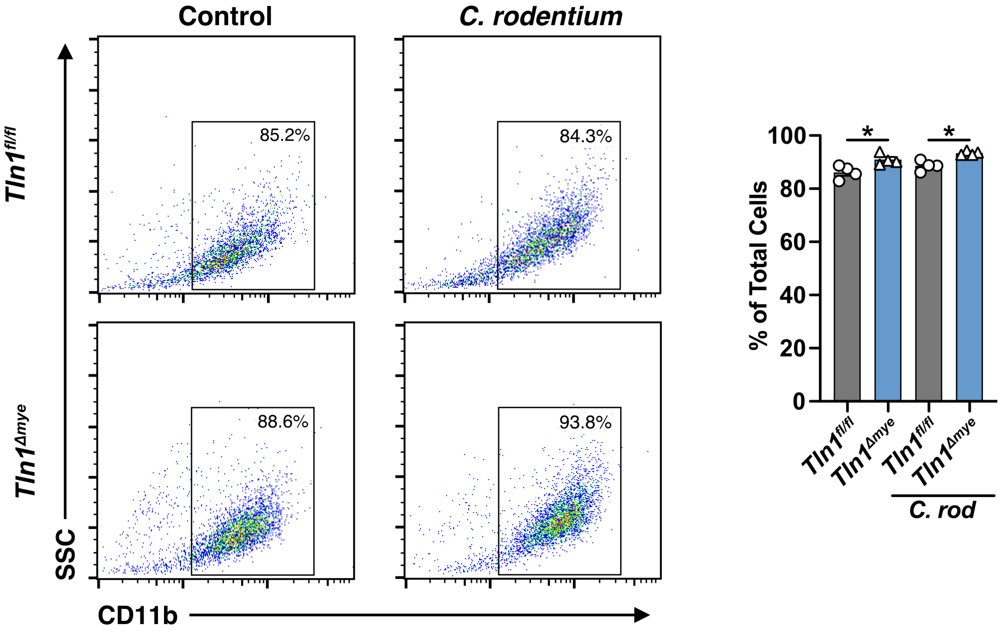

Figure S4. Surface expression of CD11b. Representative flow plots and graph depicting the surface expression and percent of CD11b-expressing BMmacs derived from *Tln1*$^{fl/fl}$ and *Tln1*^{mye} mice. All values are reported as mean ± SEM. **P*<0.05 determined by 1-way ANOVA and Tukey post hoc test.

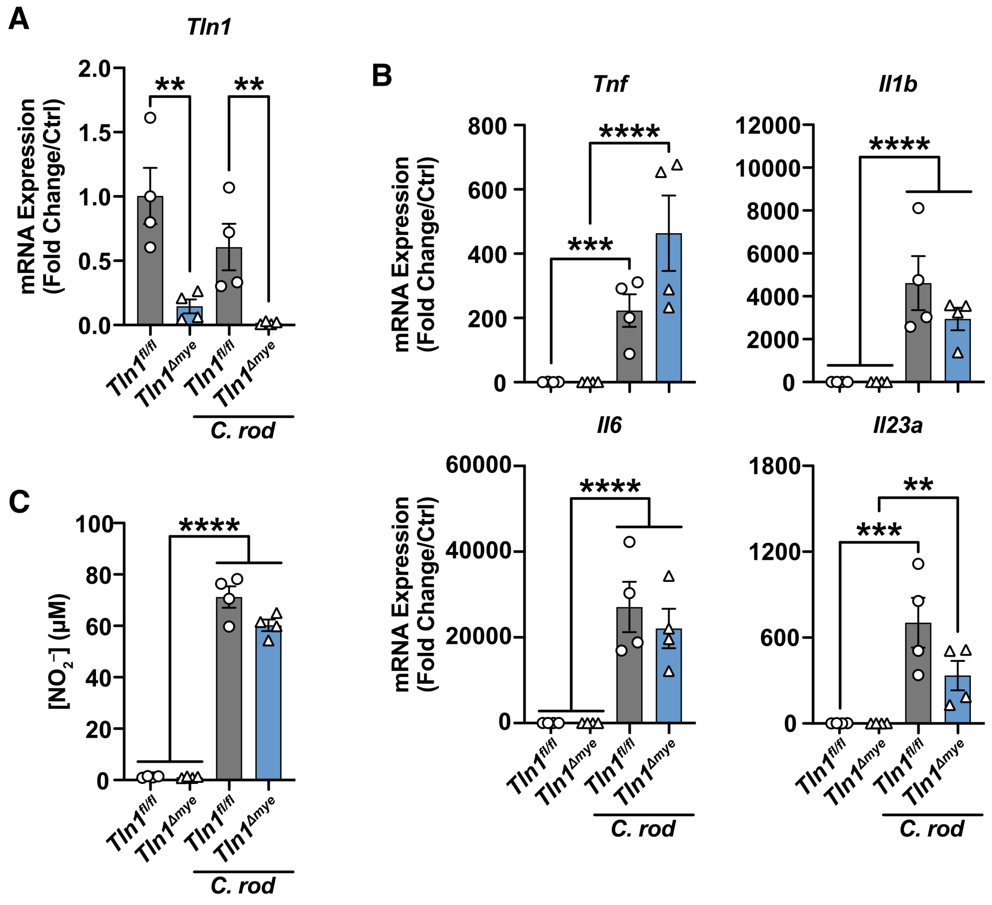

Figure S5. Talin-1 does not contribute to dendritic cell activation. BMDCs derived from *Tln1*$^{fl/fl}$ and *Tln1*$^{\Delta mye}$ mice were infected or not with *C. rodentium*, $n = 4$ mice per genotype. (**A**) Expression of *Tln1* mRNA determined by qRT-PCR 6 h post-infection. (**B**) Expression of pro-inflammatory genes at 6 h post-infection. (**C**) The concentration of NO_2^- in cell supernatants 24 h post-infection measured by the Griess reaction. All values are reported as mean ± SEM. Statistical analyses, where shown; *P<0.05, **P<0.01, ***P<0.001, and ****P<0.0001 determined by 1-way ANOVA and (**B**) Tukey post hoc test or (**C**) Kruskal-Wallis test, followed by Mann-Whitney U tests.

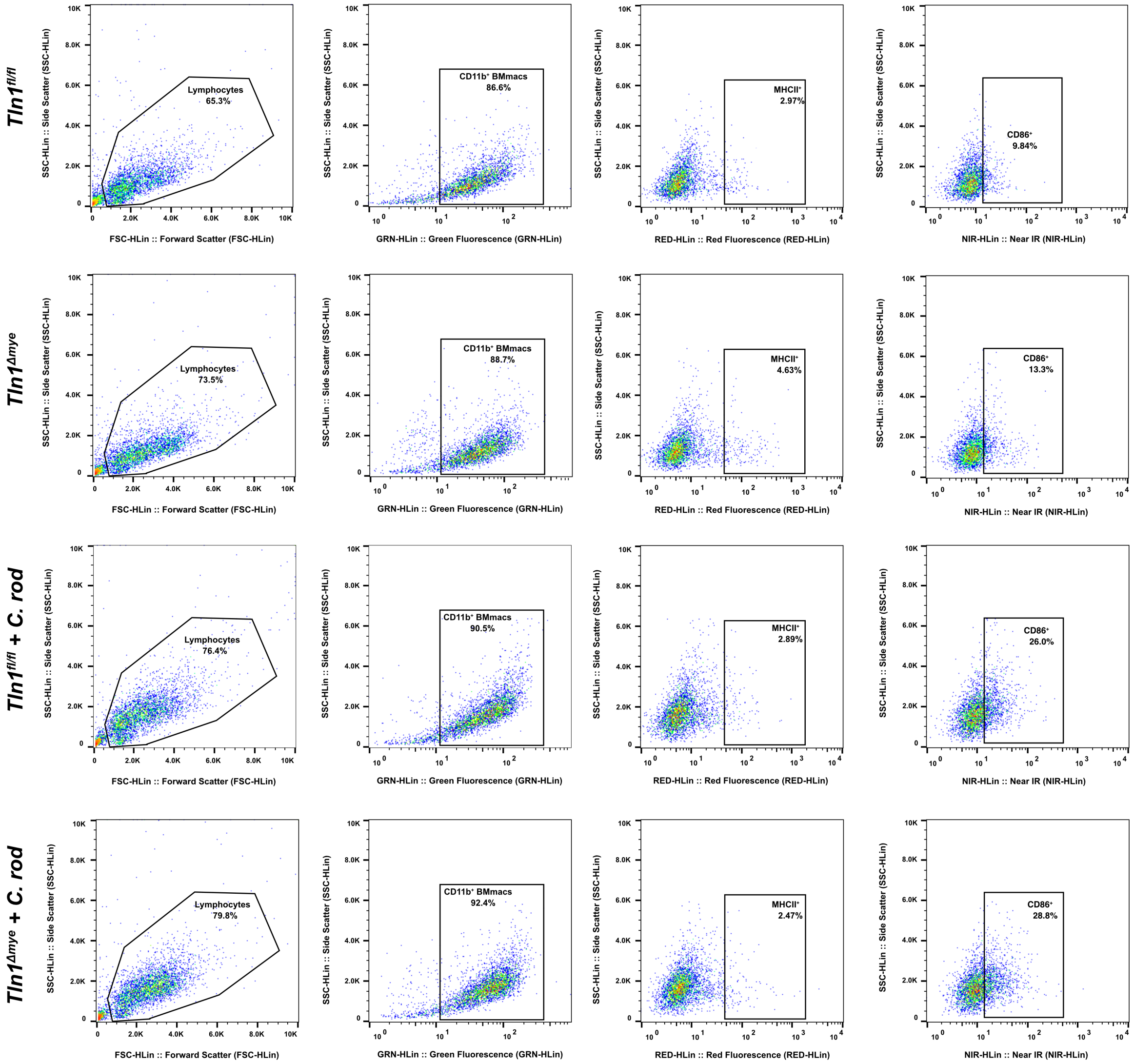

Figure S6. Gating strategy for expression of MHCII and CD68. Representative flow plots depicting the surface expression and percentage of MCHII and CD68 on CD11b$^+$ BMmacs derived from uninfected and infected *Tln1$^{fl/fl}$* and *Tln1^{mye}* mice.

Table S1: List of primers used for this paper

Target gene	Sequence (5'-3')
Tln1	F: GGCCCTCCCAACGACTTT
	R: AGCCTCTAGCCAGATGCCTTT
Tnf	F: CTGTGAAGGGAATGGGTGTT
	R: GGTCACTGTCCCAGCATCTT
Il1b	F: ACCTGCTGGTGTGTGACGTTCC
	R: GGGTCCGACAGCACGAGGCT
Il6	F: AGTTGCCTTCTTGGGACTGA
	R: TCCACGATTTCCCAGAGAAC
Il23a	F: CCAGCAGCTCTCTCGGAATC
	R: TCATAGTCCCGCTGGTGC
Cxcl10	F: GGTCTGAGTGGGACTCAAGG
	R: GTGGCAATGATCTCAACACG
Arg1	F: AAGAAAAGGCCGATTCACCT
	R: CACCTCCTCTGCTGTCTTCC
Tnfsf14	F: CTGCATCAACGTCTTGGAGA
	R: GATACGTCAAGCCCCTCAAG
Ifng	F: GGCCATCAGCAACAACATAAGCGT
	R: TGGGTTGTTGACCTCAAACTTGGC
Il17a	F: ATCCCTCAAAGCTCAGCGTGTC
	R: GGGTCTTCATTGCGGTGGAGAG
Il22	F: TTGAGGTGTCCAACTTCCAGCA
	R: AGCCGGACGTCTGTGTTGTTA
Actb	F: CCAGAGCAAGAGAGGTATCC
	R: CTGTGGTGGTGAAGCTGTAG
Il10	F: CCAAGCCTTATCGGAAATGA
	R: TCACTCTTCACCTGCTCCAC
Tgfb1	F: TCCTTGCCTGCGGAAGT
	R: GGAGAGCATTGAGCAGTTCGA

Table S2. List of antibodies used for the monocyte-macrophage waterfall assay.

Antigen-label	Manufacturer	Catalog number	Dilution
Live/Dead-eFluor506	Thermo Fisher Scientific	65-086614	1:1200
CD45-SparkYG580	Biolegend	103171	1:500
CD3-BV750	Biolegend	100373	1:400
CD4-Apc-Cy7	Biolegend	100414	1:1000
CD8-Sparkblue550	Biolegend	100780	1:800
NK1.1-FITC	Biolegend	108706	1:200
CD19-FITC	Biolegend	152404	1:1000
MHCII-PE-Cy7	Biolegend	107629	1:2000
CD11c-BV421	Biolegend	117330	1:150
Ly6C-PerCP/Cy5.5	Biolegend	128012	1:1000
Ly6G-APC	Biolegend	127613	1:500
CD11b-PerCP	Biolegend	101229	1:1500
CD103-PE	Biolegend	121406	1:400
CD64-BV605	Biolegend	139323	1:1000
CX3CR1- ApcFire810	Biolegend	149053	1:1000
